# Assessment of a Smartphone-Based Loop-Mediated Isothermal Amplification Assay for Detection of SARS-CoV-2 and Influenza Viruses

**DOI:** 10.1001/jamanetworkopen.2021.45669

**Published:** 2022-01-28

**Authors:** Douglas M. Heithoff, Lucien Barnes, Scott P. Mahan, Gary N. Fox, Katherine E. Arn, Sarah J. Ettinger, Andrew M. Bishop, Lynn N. Fitzgibbons, Jeffrey C. Fried, David A. Low, Charles E. Samuel, Michael J. Mahan

**Affiliations:** 1 Department of Molecular, Cellular, and Developmental Biology, University of California, Santa Barbara, Santa Barbara; 2 Institute for Collaborative Biotechnologies, University of California, Santa Barbara, Santa Barbara; 3 Department of Medical Microbiology and Immunology, School of Medicine, University of California, Davis, Davis; 4 Department of Materials and Department of Mechanical Engineering, University of California, Santa Barbara, Santa Barbara; 5 Department of Medical Education, Santa Barbara Cottage Hospital, Santa Barbara, California; 6 Division of Infectious Diseases, Santa Barbara Cottage Hospital, Santa Barbara, California; 7 Department of Pulmonary and Critical Care Medicine, Santa Barbara Cottage Hospital, Santa Barbara, California

## Abstract

**Importance:**

A critical need exists in low-income and middle-income countries for low-cost, low-tech, yet highly reliable and scalable testing for SARS-CoV-2 virus that is robust against circulating variants.

**Objective:**

To assess whether a smartphone-based assay is suitable for SARS-CoV-2 and influenza virus testing without requiring specialized equipment, accessory devices, or custom reagents.

**Design, Setting, and Participants:**

This cohort study enrolled 2 subgroups of participants (symptomatic and asymptomatic) at Santa Barbara Cottage Hospital. The symptomatic group consisted of 20 recruited patients who tested positive for SARS-CoV-2 with symptoms; 30 asymptomatic patients were recruited from the same community, through negative admission screening tests for SARS-CoV-2. The smartphone-based real-time loop-mediated isothermal amplification (smaRT-LAMP) was first optimized for analysis of human saliva samples spiked with either SARS-CoV-2 or influenza A or B virus; these results then were compared with those obtained by side-by-side analysis of spiked samples using the Centers for Disease Control and Prevention (CDC) criterion-standard reverse transcriptase–quantitative polymerase chain reaction (RT-qPCR) assay. Next, both assays were used to test for SARS-CoV-2 and influenza viruses present in blinded clinical saliva samples obtained from 50 hospitalized patients. Statistical analysis was performed from May to June 2021.

**Exposures:**

Testing for SARS-CoV-2 and influenza A and B viruses.

**Main Outcomes and Measures:**

SARS-CoV-2 and influenza infection status and quantitative viral load were determined.

**Results:**

Among the 50 eligible participants with no prior SARS-CoV-2 infection included in the study, 29 were men. The mean age was 57 years (range, 21 to 93 years). SmaRT-LAMP exhibited 100% concordance (50 of 50 patient samples) with the CDC criterion-standard diagnostic for SARS-CoV-2 sensitivity (20 of 20 positive and 30 of 30 negative) and for quantitative detection of viral load. This platform also met the CDC criterion standard for detection of clinically similar influenza A and B viruses in spiked saliva samples (n = 20), and in saliva samples from hospitalized patients (50 of 50 negative). The smartphone-based LAMP assay was rapid (25 minutes), sensitive (1000 copies/mL), low-cost (<$7/test), and scalable (96 samples/phone).

**Conclusions and Relevance:**

In this cohort study of saliva samples from patients, the smartphone-based LAMP assay detected SARS-CoV-2 infection and exhibited concordance with RT-qPCR tests. These findings suggest that this tool could be adapted in response to novel CoV-2 variants and other pathogens with pandemic potential including influenza and may be useful in settings with limited resources.

## Key Points

**Question:**

Can loop-mediated isothermal amplification (LAMP)-based methodology coupled with smartphone detection provide an inexpensive, rapid, sensitive, and reliable platform for COVID-19 and influenza testing?

**Findings:**

In this cohort study of saliva samples from 50 community-based patients, the smartphone-based LAMP assay detected SARS-CoV-2 infection and exhibited concordance with reverse transcriptase–quantitative polymerase chain reaction tests.

**Meaning:**

These findings suggest that the smartphone-based LAMP assay offers an additional tool to detect COVID-19 that can be readily modified in response to novel SARS-CoV-2 variants and other pathogens with pandemic potential including influenza.

## Teaser

This cohort study examines whether a smartphone-based loop-mediated isothermal amplification (LAMP) assay is suitable for SARS-CoV-2 and influenza virus testing without requiring specialized equipment, accessory devices, or custom reagents.

## Introduction

The SARS-CoV-2 virus responsible for the COVID-19 pandemic has infected more than 294 million people resulting in more than 5.5 million deaths worldwide, including many in countries lacking technical and financial resources to effectively monitor and respond to the pandemic.^1^ Accordingly, in addition to immunization of vulnerable populations, there is an urgent need for simple, accurate, and low-cost testing platforms at the point-of-care that can be used by health care clinicians and other authorities in remote and resource-limited settings.^2^ Numerous methods for the detection of SARS-CoV-2 virus, including molecular, antigen, and serology tests, are currently in use.^3,4,5,6,7,8^ Although molecular methods such as polymerase chain reaction (PCR) are rapid and sensitive, they generally require access to specialized and costly laboratory instrumentation and highly trained personnel, and they are technologically complex for point-of-care (POC) applications or resource-limited settings. Although antigen and serology tests are simple to use, cost-effective and portable, they can be unreliable with high false-positive and false-negative rates due to their lack of sensitivity. High-throughput sequencing-based approaches have gained broad utility for detection and genotyping of emerging CoV-2 variants, but they do require RNA extraction and expensive thermocycling and sequencing devices.^9,10,11,12^

Loop-mediated isothermal amplification (LAMP) diagnostics have gained attention for pathogen detection because they do not require sophisticated, expensive instrumentation or highly trained personnel for operation.^13^ However, the high sensitivity and utility of LAMP-based diagnostics historically has been offset by a propensity for primer-dimer self-amplification owing to the requirement of 6 primers per target gene, potentially increasing the incidence of false positives.^14,15,16^ We aimed to address this problem by determining experimental conditions that effectively eliminate primer-dimer amplification, thereby permitting the development of a highly sensitive LAMP-based test for SARS-CoV-2 and influenza A and B viruses. Indeed, the highly similar clinical symptoms of SARS-CoV-2 and influenza has prompted the US Centers for Disease Control and Prevention (CDC) and the World Health Organization recommendations for combination diagnostics when both pathogens are circulating.^17,18,19^ Furthermore, the lifting of pandemic restrictions exacerbates the potential for a dual epidemic of COVID-19 and influenza, termed a perfect storm, because of a potential increase in severe illness, transmission, and misdiagnosis resulting from symptom overlap.^20,21,22,23^

Five principal tenets of POC clinical diagnostics include speed, sensitivity, affordability, scalability, and accessibility, without a concomitant need of specialized and costly equipment. Although a number of innovative systems have been described for the detection of SARS-CoV-2, current systems are lacking in one or more of the diagnostic parameters necessary for implementation at the POC. Examples include the CDC 2019-nCoV reverse transcriptase–quantitative polymerase chain reaction (RT-qPCR) test that is sensitive but requires expensive instrumentation^24^; and, the CDC influenza-SARS-CoV-2 combination diagnostic (Flu SC2 RT-qPCR multiplex assay) that requires custom probes that are expensive and susceptible to supply chain availability.^25^ Although the Abbott ID NOW test is rapid, sensitive, and uses isothermal amplification, it requires specialized reagents and instrumentation with limited scalability and availability.^26^ The SalivaDirect test is an extraction-free protocol using saliva, but it involves RT-PCR amplification and hence is subject to similar limitations as the CDC tests for POC applications.^27^

Smartphones are ideally suited to meet the need for low-cost, widely accessible clinical POC diagnostic tools, with smartphone global use estimated at nearly half the world’s population.^28^ The potential to transform mobile phones into clinical POC diagnostic tools is evidenced by a number of detection modalities, including those based on optical and fluorescence imaging, microfluidic chips, biosensors, microelectronics, lateral flow, and nucleic acid or immunologic detection.^29,30,31,32,33,34,35,36,37,38,39,40^ However, these innovative systems generally require the coupling of the mobile phone to specialized accessory diagnostic devices, custom reagents, or sophisticated protocols. Examples include a LAMP system that uses a 3-D cartridge and a smartphone-based reader^37^; a centrifugal microfluidic platform with a smartphone read-out^39^; and a CRISPR-Cas13a assay that features mobile phone microscopy.^38^

In this study, we investigate a low-tech test, termed smartphone-based real-time loop-mediated isothermal amplification (smaRT-LAMP), to see whether it possesses the combined attributes of speed, sensitivity, low cost, scalability, and accessibility for rapid and frequent POC testing.

## Methods

Human participant approval was obtained from the Institutional Human Subjects Use Committee of the University of California, Santa Barbara, and the institutional review board of Santa Barbara Cottage Hospital. Written informed consent was obtained from all participants. An in-person or remote video interpreter was used as needed. This cohort study followed the Standards for Reporting of Diagnostic Accuracy (STARD) reporting guideline. Race and ethnicity were not considered in this study.

### LAMP Protocol Advances That Increase Sensitivity and Eliminate False Positives Due to Primer-Dimer Amplification

The clinical utility of LAMP-based diagnostics have been limited by false positives (primer-dimer amplification).^14,15,16^ This technical challenge was overcome by the development of a LAMP protocol that optimizes experimental conditions for viral RNA stability and cDNA synthesis, which both increased sensitivity and effectively eliminated false positives due to primer-dimer amplification (eMethods, eTable 1, eFigure 1, and eFigure 2 in the Supplement). We found that in addition to reaction mixture composition, the order of assembly of the reaction mixture components was critical to improve LAMP performance and reduce primer-dimer formation. Furthermore, the smartphone app imparts a 25-minute reaction time cutoff to distinguish specimen-sample amplification (early) from primer-dimer amplification (late).

### Study Design

A head-to-head comparison of smaRT-LAMP, hereafter referred to as smartphone-based LAMP assay, and criterion-standard RT-qPCR methodologies was performed using human saliva samples spiked with either SARS-CoV-2 or influenza viruses. These analyses were then used as the basis for development of a smartphone-based LAMP detection platform for SARS-CoV-2 from self-collected clinical saliva samples obtained from hospitalized patients with COVID-19. Sensitivity and specificity tests using spiked saliva specimens were performed as per the Food and Drug Administration’s Emergency Use Authorization guidelines^41^ (eMethods in the Supplement).

Participants were deemed eligible based on the STARD reporting guideline for diagnostic tests.^42^ Eligible participants had no prior known SARS-CoV-2 infection, and presented with a new positive or negative SARS-CoV-2 PCR test, obtained within 12 hours of specimen collection. Potential participants were identified by the presence of new flu-like symptoms or dyspnea in the hospital emergency department or an inpatient ward. Recruitment took place between January 12, 2021, and May 11, 2021, and were enrolled forming a convenience series.

Clinical saliva samples were evaluated for SARS-CoV-2, influenza A and B sensitivity, and quantitative detection of viral load. Fifty patient saliva specimens were split into equal volumes and a head-to-head comparison of the smartphone-based LAMP assay with the CDC RT-qPCR assay was performed. Sensitivity was determined by the presence or absence of sample signal (binary plus or minus call). Viral loads were determined by comparison of sample signal with that of standard curves established by serial dilution of spiked saliva samples (SARS-CoV-2) or by relative TCID_50_ quantitation (50% tissue culture infective dose assay) of spiked saliva samples (influenza A and B) (eMethods in the Supplement).

### Strains

The following virus strains and gRNAs were used as described in the eMethods in the Supplement: (1) Coronaviruses: SARS-CoV-2, USA-WA, and Hong Kong isolates; human seasonal coronaviruses HCoV-OC43, HCoV-229E, HCoV-NL63, and HCoV-HKU1; SARS-CoV-1; and MERS-CoV; (2) SARS-CoV-2 variant gRNAs: Alpha, B.1.1.7 (UK); Gamma, P.1 (Brazil); Delta, B.1.617.2 (India); Epsilon, B.1.429 (CAL20C); and Iota, B.1.526 (NY); (3) influenza virus strains: influenza A (H1N1) and influenza B (Yamagata); (4) bacterial respiratory pathogens: *Streptococcus pneumoniae* D39, *Staphylococcus aureus* USA 300, *Pseudomonas aeruginosa* ATCC 10145, and *Klebsiella pneumoniae* ATCC 13883.

### Gene Targets

The smartphone-based LAMP assay primers target the SARS-CoV-2 nucleocapsid (N) and ORF1ab genes; influenza primers target genes encoding matrix protein (M1) and polymerase (PB1) for influenza A; and M1 and nonstructural protein (NS1) for influenza B (eTable 1 in the Supplement). Primers for CDC 2019-nCoV RT-qPCR analysis target the SARS-CoV-2 N gene,^24^ whereas the influenza SARS-CoV-2 (Flu SC2) RT-qPCR multiplex assay targets M1 for influenza A and NS2 for influenza B.^25^

### Statistical Analyses

The smartphone-based LAMP assay and RT-qPCR molecular diagnostic sensitivity and specificity were determined by comparing the proportion of samples that amplified with cognate vs noncognate primers (or no template), using the Mantel–Haenszel test (two-tailed). *P* values of less than .05 were considered significant. The exact value of n, representing the number of independent biological determinations, was indicated in the figure legends. Statistical analysis was performed using Epicalc 2000 version 1.02 (Brixton Books) from May to June 2021.

## Results

Of the study’s 50 eligible participants with no prior SARS-CoV-2 infection, 29 were men. The mean age was 57 years (range, 21 to 93 years).

### Sensitivity and Specificity of Smartphone-Based LAMP Assay Platform

#### SARS-CoV-2 Sensitivity

**Figure 1.**
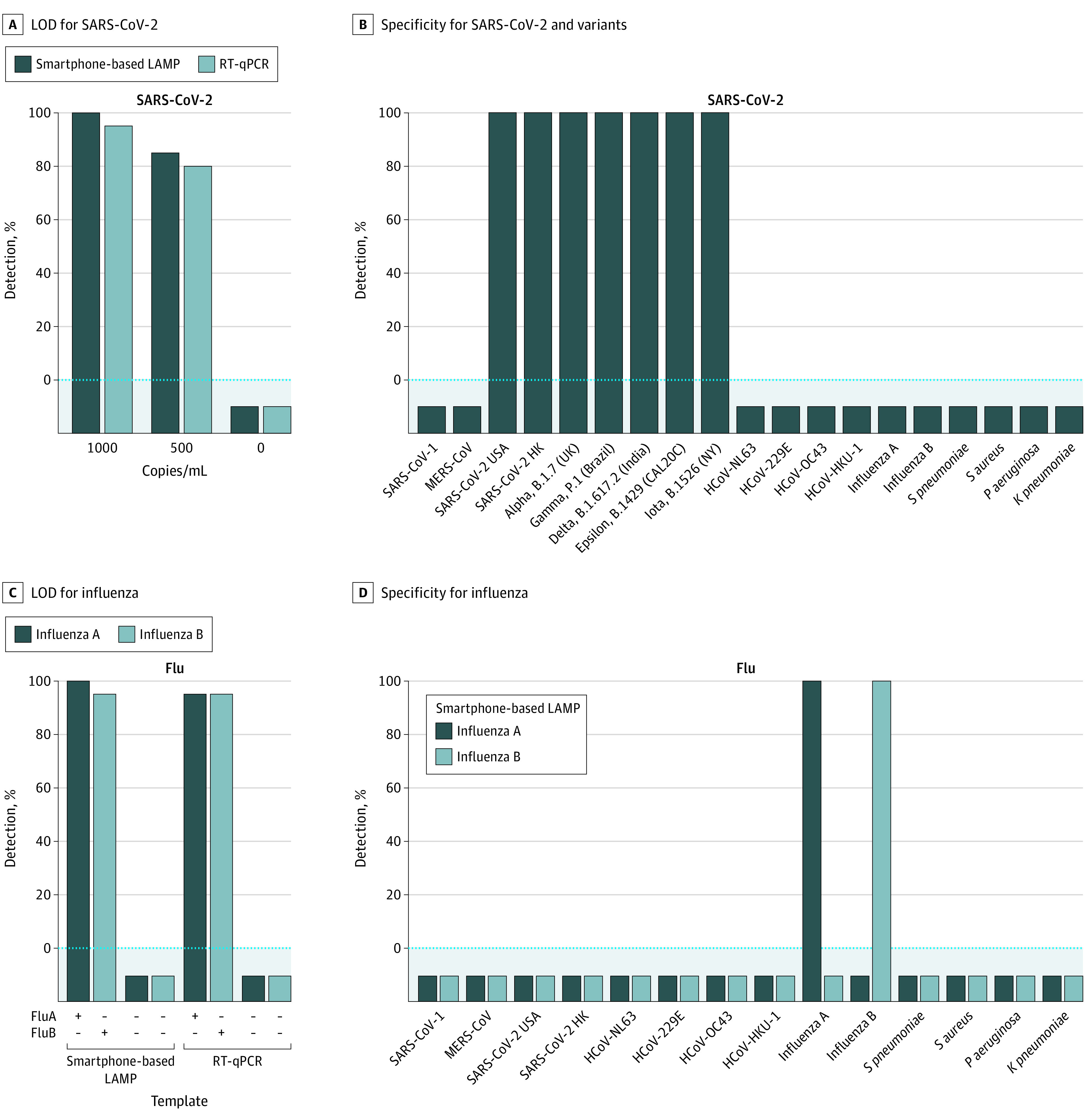
Smartphone-Based LAMP Assay Sensitivity and Specificity for SARS-CoV-2 and Influenza Viruses A, Limit of detection (LOD) for SARS-CoV-2 measured by the smartphone-based loop-mediated isothermal amplification (LAMP) assay vs Centers for Disease Control and Prevention (CDC) 2019-nCoV reverse transcriptase–quantitative polymerase chain reaction (RT-qPCR), using SARS-CoV-2 primers, was determined by the largest serial dilution of SARS-CoV-2 USA viral stock giving a signal in at least 19 of 20 biological replicates. B, Specificity (cross-reactivity) of smartphone-based LAMP assay for SARS-CoV-2, CoV-2 variants, and other viral and bacterial respiratory pathogens, using SARS-CoV-2 primers was determined by the presence or absence of signal (binary plus or minus call) (eMethods in the Supplement). C, LOD for influenza A and B measured by smartphone-based LAMP vs CDC Flu SC2 RT-qPCR was determined as in (A) using influenza A or B primers. D, Specificity was evaluated as in (B) using influenza A and B primers. n = 10 biological replicates for SARS-CoV-2 variants; n = 20 biological replicates for all other pathogens.

A comparative limit-of-detection (LOD) analysis of the smartphone-based LAMP assay platform vs the clinical criterion standard CDC 2019-nCoV RT-qPCR diagnostic^24^ was carried out using spiked saliva samples analyzed in parallel (Figure 1A). The LOD of the smartphone-based LAMP assay for SARS-CoV-2 was 10^3^ copies/mL, matching that of the CDC 2019-nCoV RT-qPCR diagnostic test (20 of 20 and 19 of 20 biological replicates, respectively). The saliva samples from virus-negative donors, when not spiked, gave no amplification (0 of 20 biological replicates; *P* < .001).

#### SARS-CoV-2 Specificity

The smartphone-based LAMP assay specificity for SARS-CoV-2 was evaluated by measuring cross-reactivity against several different viral and bacterial respiratory pathogens (Figure 1B). The smartphone-based LAMP assay using SARS-CoV-2 primers amplified both of the early SARS-CoV-2 isolates tested (USA-WA and Hong Kong) (20 of 20 biological replicates). No amplification was observed for any of the other 6 coronaviruses tested (SARS-CoV-1, MERS-CoV, HCoV-OC43, HCoV-229E, HCoV-NL63, and HCoV-HKU1). Neither influenza A or B nor any of the 4 bacterial respiratory pathogens tested (*S pneumoniae*, *S aureus, P aeruginosa*, and *K pneumoniae*) were detected with the SARS-CoV-2 primers (0 of 20 biological replicates; *P* < .001) (Figure 1B). Taken together, the smartphone-based LAMP assay was rapid (25 minutes), sensitive (1000 copies/mL), and could simultaneously analyze up to 96 samples per phone, at a cost of less than $7 per test (Figure 1A; eTable 3 and eFigure 2 in the Supplement).

#### SARS-CoV-2 Variants

SARS-CoV-2 variants continue to surge throughout the world and thus it is critical that molecular diagnostics are able to accurately detect them.^43^ The smartphone-based LAMP assay detected genomic RNA isolated from 5 of 5 major SARS-CoV-2 variants tested: Alpha, B.1.1.7 (UK); Gamma, P.1 (Brazil); Delta, B.1.617.2 (India); Epsilon, B.1.429 (CAL20C); and Iota, B.1.526 (NY) (10 of 10 biological replicates; *P* < .001) (Figure 1B). None of the base alterations present in these variants overlapped with any of the smartphone-based LAMP assay primer sequences. Thus, it was not unexpected that the base alterations did not affect smartphone-based LAMP detection of the variants (eTable 2 in the Supplement).

#### Influenza A and B Sensitivity

A comparative LOD analysis of the smartphone-based LAMP assay platform vs the clinical criterion-standard CDC influenza SARS-CoV-2 (Flu SC2) RT-qPCR multiplex assay^25^ also was evaluated using spiked saliva samples and either influenza A or influenza B primers (Figure 1C). The LOD of smartphone-based LAMP assay matched that of Flu SC2 RT-qPCR test for influenza A (2.8 × 10^2^ TCID_50_/mL) and exceeded that for influenza B (0.8 vs 40 TCID_50_/mL) (19 of 20 biological replicates). Unspiked saliva samples from virus-negative donors gave no amplification with either influenza A or B primers (0 of 20 biological replicates; *P* < .001).

#### Influenza A and B Specificity

Smartphone-based LAMP assay specificity for influenza A or B was evaluated as previously mentioned using either influenza A or B primers with spiked saliva samples (Figure 1D). The smartphone-based LAMP assay amplified influenza A or B viruses when using cognate influenza A or B primers (20 of 20 biological replicates), whereas no amplification was observed with any of the 8 coronaviruses or 4 bacterial pathogens tested (0 of 20 biological replicates; *P* < .001).

### Clinical Evaluation

**Figure 2.**
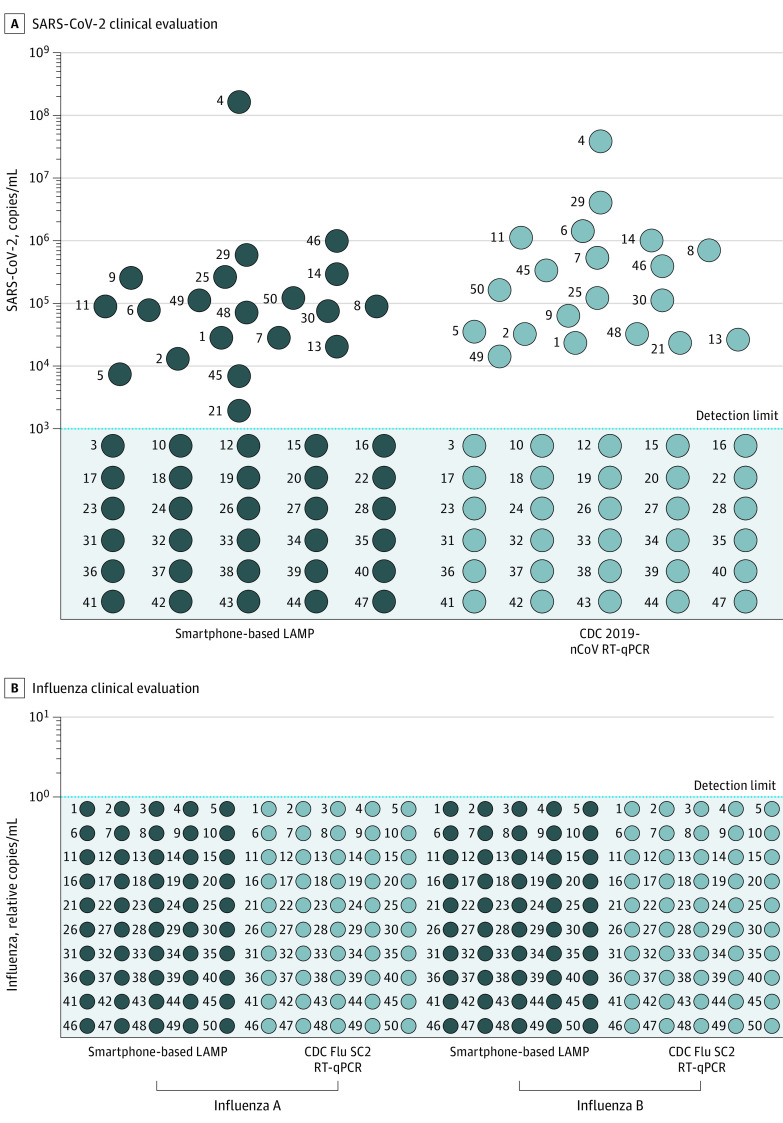
Clinical Evaluation of SARS-CoV-2 Patient Saliva Specimens Fifty patient saliva specimens were split into equal volumes and a comparative analysis was performed for (A) SARS-CoV-2 using the smartphone-based loop-mediated isothermal amplification (LAMP) assay or Centers for Disease Control and Prevention (CDC) 2019-nCoV reverse transcriptase–quantitative polymerase chain reaction (RT-qPCR); and for (B) influenza A or B using smartphone-based LAMP assay or CDC Flu SC2 RT-qPCR assay. Sensitivity was determined by the presence or absence of sample signal (binary plus or minus call). Quantitative detection of SARS-CoV-2 was determined by comparison of sample signal with that of standard curves established from serial dilution of spiked saliva samples amplified with the smartphone-based LAMP assay or CDC 2019-nCoV RT-qPCR. Influenza A and B copy number was determined by relative TCID_50_ quantitation (50% tissue culture infective dose assay) of spiked saliva samples (eMethods in the Supplement).

Saliva samples were obtained from 50 eligible participants with no prior SARS-CoV-2 infection (age range, 21 to 93 years; mean age, 57 years; 21 female participants, 29 male participants; recruitment took place January 12, 2021, to May 11, 2021). The smartphone-based LAMP assay showed 100% concordance with the RT-qPCR diagnostic for SARS-CoV-2 sensitivity (20 of 20 positive and 30 of 30 negative) and for quantitative detection of viral load (copies/mL) (Figure 2A). The smartphone-based LAMP assay also matched the performance of RT-qPCR assays for influenza A and B sensitivity and viral load (50/50 negative) (Figure 2B), consistent with CDC reports that influenza cases were low during the 2020-2021 season, possibly due to COVID-19 mitigation by face masking and social distancing restrictions.^44^

### Storage Conditions for Patient Saliva Specimens

**Figure 3.**
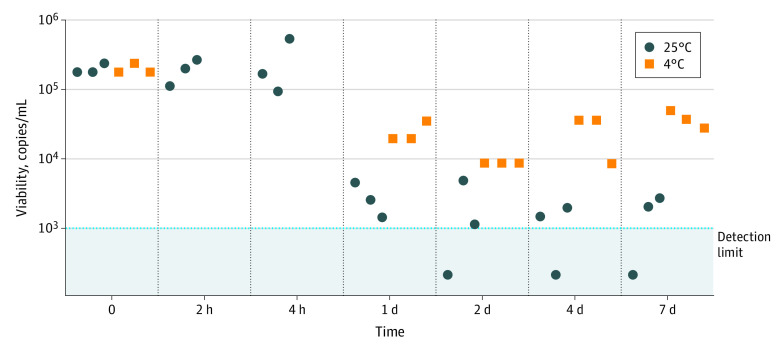
Smartphone-Based LAMP Assay Compatibility With Room Temperature Specimen Storage and Reaction Assembly Spiked saliva samples (2.5 × 10^5^ copies/mL) were prepared in fresh saliva from virus-negative donors. The viral concentration (copies/mL) as a function of time and temperature was evaluated by the smartphone-based LAMP assay using a reaction mix assembled at room temperature. Quantitative detection of viral load was determined by comparison of sample signal with that of a standard curve established from serial dilution of spiked saliva samples amplified with smartphone-based LAMP assay; n = 3 biological replicates for each condition.

Smartphone-based LAMP assay compatibility with room temperature storage of saliva specimens and reaction mix assembly for SARS-CoV-2 testing was evaluated to determine suitability for use in resource-limited settings wherein refrigeration may not be readily available. Viral concentration was determined as a function of storage time and temperature by the smartphone-based LAMP assay, using reaction mixes assembled at room temperature. The smartphone-based LAMP assay amplified SARS-CoV-2 contrived samples without substantial loss of sensitivity after sample storage for less than or equal to 4 hours at room temperature and less than 10-fold loss in sensitivity after sample storage for up to at least 1 week at 4 °C (Figure 3).

## Discussion

COVID-19 pandemic control efforts require accurate, accessible diagnostic testing that is robust against the circulating SARS-CoV-2 variants. As a step toward achieving this goal, we developed a smartphone-based LAMP assay. This simple, low-tech test is rapid, sensitive, low cost, and scalable without the need for specialized and costly equipment. A head-to-head comparison revealed that the smartphone-based LAMP assay matched the performance of the clinical criterion-standard RT-qPCR diagnostic test for SARS-CoV-2 sensitivity and quantitative detection of viral load. This suggests that the smartphone-based LAMP assay is a reliable test for SARS-CoV-2 virus. This system used LAMP protocol advances that optimized experimental conditions for viral RNA stability and cDNA synthesis, which increased sensitivity and effectively eliminated false positives due to primer-dimer amplification. The LAMP protocol described in this study has the potential to facilitate LAMP-based diagnostic tests for other pathogens in addition to SARS-CoV-2 and influenza viruses. The smartphone-based LAMP assay of saliva specimens performed well under room temperature conditions, and circumvented the need for expensive fluorescent probes. It may be particularly useful in the context of settings that may otherwise lack sophisticated instrumentation, specialized reagents, or suitably trained personnel.

The smartphone-based LAMP assay detection system consists of a hot plate, cardboard box, and LED lights. The system is inexpensive to set up and portable; it can be fabricated for less than $100 (in addition to the smartphone cost, which is approximately $200 used or approximately $400 new). The smartphone-based LAMP assay thus offers the potential to leverage a readily accessible technology to inexpensively deliver state-of-the-art nucleic acid diagnostics for quantitative pathogen detection at the POC. As reported in this study and by others, SARS-CoV-2 can be detected in saliva samples.^27,45,46^ Saliva testing has numerous advantages relative to nasopharyngeal swabs, including cost and ease of use (self-collection vs trained personnel and PPE for sample collection). Furthermore, saliva sampling circumvents the need for specialized swabs and reagents, test components that are vulnerable to supply-chain disruptions and resource limitations.

There are opportunities to adapt our protocol from a lab test to a field test and to further reduce the cost of smartphone-based LAMP diagnostics. First, because approximately 90% of the cost per test comes from using commercially available enzymes, bulk enzyme purification or purchase could further lower the cost substantially. Second, field-test applicability could conceivably be achieved with the use of lyophilized reagents, which would be well-suited for areas that lack refrigeration.^47,48^ The use of lyophilized reagents can further streamline the LAMP assay by enabling the addition of specimen directly to a preassembled master mix, thereby minimizing sample handling, preparation time and the potential for user error, while enhancing user biosafety. Additionally, the system provides a platform for inexpensive home-based testing.

The capacity to rapidly and accurately test large populations, including individuals in developing nations struggling with inadequate vaccine supplies and testing access amidst a landscape of new, and more highly transmissible variants, is critically important. Accordingly, the smartphone-based LAMP assay detected 5 of 5 major SARS-CoV-2 variants including Alpha and Delta. Through primer changes, the LAMP assay could be further adjusted as necessary if new, as yet unseen, variants evolve. The Omicron variant, which emerged during the manuscript review process and possesses approximately 50 variations with many clustered in the gene encoding the spike protein,^49^ would not be expected to escape diagnostic detection of the LAMP primers described in this study. The broad applicability of a smartphone-based assay has been previously demonstrated with excellent performance for urinary bacterial diagnostics and for the detection of multiple microbial pathogens in body fluids (blood, urine, feces).^50^ The LAMP assay now potentially offers underserved populations with an additional testing tool for the next stage in the pandemic. Moreover, the lifting of COVID pandemic restrictions is expected to markedly increase influenza cases during the upcoming flu season and has prompted CDC recommendations for SARS-CoV-2 and influenza combination diagnostics when both pathogens are circulating.^17,51^ Finally, integration of the smartphone-based LAMP assay with telemedicine has the potential to deliver advanced health care to vulnerable populations, while markedly broadening the scope of personalized medicine.^52,53^ Taken together, the LAMP assay integrates state-of-the-art diagnostic techniques with the connectivity and computational power of the smartphone, offering the potential to provide fair and equal access to precision diagnostic medicine.

### Limitations

This study had some limitations. Although the goal of the study was to develop a smartphone-based test suitable for low-income and middle-income countries, the study participants were hospitalized patients in the United States and, thus, the experimental outcome under low-income and middle-income country conditions is yet to be determined. Furthermore, the study could be improved with an increased number of participants (symptomatic and asymptomatic) beyond the current 50, which limits the interpretation of the data.

## Conclusions

The smartphone-based LAMP assay integrates reliable diagnostics with advantages of smartphone detection, offering an inexpensive diagnostic platform for SARS-CoV-2 and influenza A and B viruses that match the CDC RT-qPCR criterion standards. The capacity to rapidly and accurately test saliva samples from populations presently lacking adequate vaccination levels and access to viral testing amidst the emergence of more highly transmissible variants is critically important. Moreover, as new variants of SARS-CoV-2 virus emerge, testing and detection remain at the forefront of pandemic control efforts. The smartphone-based LAMP assay thus offers the potential to provide a critical tool to mitigate further stages of the COVID-19 pandemic. Additionally, the LAMP assay can be readily engineered to address novel CoV-2 variants and other pathogens with pandemic potential, including influenza.

## Notes

Supplement. **eMethods.** Supplemental Materials **eTable 1.** Oligonucleotide Primer Sequences **eTable 2.** Evaluation of LAMP Primer Sequences for Nucleotide Mutations Present in SARS-CoV-2 Variants of Concern **eTable 3.** SmaRT-LAMP Test Expenditures, Equipment and Scale-up Protocol for Reaction Mix **eFigure 1.** Overview of SmaRT-LAMP Instrumentation and Workflow **eFigure 2.** Workflow of the Bacticount SmaRT-LAMP Mobile Phone App
eReferences
