# Supplementary material for: Assessment of a Smartphone-Based Loop-Mediated Isothermal Amplification Assay for Detection of SARS-CoV-2 and Influenza Viruses
Source: JAMA Netw Open. 2022 Jan 28;5(1):e2145669. doi: 10.1001/jamanetworkopen.2021.45669 (PMC8800074; doi:10.1001/jamanetworkopen.2021.45669)
Supplement: Supplement. — eMethods. Supplemental Materials eTable 1. Oligonucleotide Primer Sequences eTable 2. Evaluation of LAMP Primer Sequences for Nucleotide Mutations Present in SARS-CoV-2 Variants of Concern eTable 3. SmaRT-LAMP Test Expenditures, Equipment and Scale-up Protocol for Reaction Mix eFigure 1. Overview of SmaRT-LAMP Instrumentation and Workflow eFigure 2. Workflow of the Bacticount SmaRT-LAMP Mobile Phone App eReferences [file jamanetwopen-e2145669-s001.pdf]

Supplemental Online Content

Heithoff DM, Barnes L V, Mahan SP, et al. Assessment of a smartphone-based loop-mediated isothermal amplification assay for detection of SARS-CoV-2 and influenza viruses. *JAMA Netw Open.* 2022;5(1):e2145669. doi:10.1001/jamanetworkopen.2021.45669

eMethods. Supplemental Methods

eTable 1. Oligonucleotide Primer Sequences

eTable 2. Evaluation of LAMP Primer Sequences for Nucleotide Mutations Present in SARS-CoV-2 Variants of Concern

eTable 3. SmaRT-LAMP Test Expenditures, Equipment and Scale-up Protocol for Reaction Mix

eFigure 1. Overview of SmaRT-LAMP Instrumentation and Workflow

eFigure 2. Workflow of the Bacticount SmaRT-LAMP Mobile Phone App

eReferences

This supplemental material has been provided by the authors to give readers additional information about their work.

eMethods. Supplemental Methods

LAMP protocol increases sensitivity and eliminates false positives due to primer-dimer amplification

The LAMP protocol optimizes experimental conditions to favor viral RNA stability and cDNA synthesis, resulting in increased sensitivity and effectively eliminating false positives due to primer-dimer amplification. Parameters include: *i*) optimal primer design to reduce the probability of primer-dimer formation; *ii*) optimal reaction chemistry favoring primer binding to viral nucleic acids; *iii*) melting of primers at 70 °C before addition to master mix; and iv) addition of RNase inhibitor to stabilize viral RNA in saliva specimens. We found that in addition to reaction mixture composition, the order of assembly of the reaction mixture components was critical to improve LAMP performance and reduce primer-dimer self-amplification (false positives).

1. Primer design: SARS-CoV-2 LAMP primer sequences were derived from published reports[1,2] and/or designed and optimized with PrimerExplorer v.5.0 (http://primerexplorer.jp/lampv5e/index.html) (**eTable 1**). All candidate primers were subsequently screened for the likelihood of primer-dimer formation via Integrated DNA Technology Oligo Analyzer software (v3.1) (http://eu.idtdna.com/analyzer/Applications/OligoAnalyzer/), and primers with large negative ΔG (- 9 kcal/mol), which are associated with high primer-dimer potential, were further optimized to obtain a more-positive ΔG by base sliding or deletion, particularly within 8 bp of the 5' and 3' ends of primers.[3] Candidate primers were then screened for specificity via a comparative BLAST homology search of α coronavirus (HCoV-229E, HCoV-NL63), β coronavirus (HCoV-HKU1, HCoV-OC43, SARS-CoV-1, MERS) and influenza A (H1N1) and B (Yamagata) virus genomes in the NCBI data base.

2. Restrictive reaction chemistry: Standard manufacturer recommendations (https://www.neb.com) were modified to more restrictive conditions to diminish primer-dimer formation/amplification by reducing the reaction mix (50 μL) Mg^{2+} concentration (from 8.0 mM to 5.7 mM); addition of 40 mM Tris-HCl pH 7.5 to saliva sample mix (25 μL); and increasing reaction temperature (from 65 °C to 70 °C).

3. Primer melting: To reduce primer-dimer formation in the "master mix", primers were individually heated to 70 °C, cooled to room temperature, and added immediately prior to the addition of reverse transcriptase and DNA polymerase.

4. RNase inhibitor: To reduce RNA degradation, RNase inhibitor was added to saliva specimen samples prior to their addition to the master mix.

SmaRT-LAMP reaction mix assembly

General work flow: The smaRT-LAMP reaction mix was assembled at room temperature in 50 μL total reaction volume containing 25 μL "sample mix" (20 μL saliva specimen with RNA stabilizers), 25 μL "master mix" (containing lysis reagents, primers and polymerase enzymes) in 8-tube PCR strips or 96-well PCR plates, with optically clear lid strips (BioRad). The resultant smaRT-LAMP reaction mix (50 μL) was transferred to a 70 °C heat-block for lysis and to initiate reverse transcription and LAMP reactions. Amplification was monitored by the free, custom-built Bacticount app adapted from Barnes et al.[4] on Samsung Galaxy S7 or S9 phones (**eFigure 1**).

Specific work flow: Sample mix is comprised of 20 μL saliva and 5 μL RNase inhibitor/Tris-HCL buffer (2.5 μl 400 mM Tris-HCl, pH 7.5 [final specimen mix concentration of 40mM Tris-HCl]), 1.25 μL RNase inhibitor (40 U/μL), and 1.25 μL nuclease-free water. Master mix and sample mix volumes are scaled up 10% relative to the actual volumes need to test a given sample number. Reagent volumes for 96 samples are given in **eTable 2**.

Order and composition of "reaction mix" and assembly (50 μL).

Step 1. 5 μL of 10X isothermal amplification buffer (final reaction concentrations of 20 mM Tris-HCl, 10 mM $(NH_4)_2SO_4$, 50 mM KCl, 2 mM $MgSO_4$, 0.1% polysorbate 20, pH 8.8 @ 25°C), supplemented with 1.85 μL of 100mM $MgSO_4$ and 0.5 mL 40% polysorbate 20 [final reaction concentrations of 5.7 mM and 0.5% (w/v), respectively]).

Step 2. 7 μL deoxynucleotide triphosphates (10 mM each; 1.4 mM final reaction concentration).

Step 3. 2 μL fluorescence detection reagent: calcein and $MnCl_2$ [final reaction concentrations of 20 μM and 0.4 mM, respectively].

Step 4. 7.7 μL oligonucleotide primers (2 gene targets, using two LAMP primer sets per reaction): 0.34 μL x 2 of F3 and B3 (30 μM); 0.26 μL x 2 of FIP and BIP (300 μM); 1.3 μL x 2 of F-Loop and B-Loop (30 μM) [final reaction concentrations of 0.2 μM, 1.6 μM, and 0.80 μM, respectively].

© 2022 Heithoff DM et al. *JAMA Network Open.*

Step 5. In order to reduce primer-dimer formation, primers were heated individually at 70 ºC for 5 m and allowed to cool to room temperature; and added to master mix just prior to enzyme addition (primer dimers occur with no primer melting and earlier primer addition to master mix).

Step 6. High concentration *Bst* 2.0 WarmStart DNA polymerase was added (0.64 U/μL) together with RTx WarmStart reverse transcriptase (0.6 U/μL).

Step 7. Sample mix (25 μL) is added to master mix (25 μL) and the resultant reaction mix was transferred to a 70 ºC heat block for amplification.

Negative controls consisted of confirmed clinically-negative saliva from Cottage Hospital. For patient saliva testing, heat-treated aliquots were removed from - 80 ºC storage, thawed on ice and added to sample mix at room temperature. Specimen stability as a function of time and temperature was assessed in this study.

Bacticount application

The Bacticount mobile phone application for monitoring and analyzing the smaRT-LAMP assay was built on a Samsung Galaxy S7 and S9 phone and can be downloaded and installed free of charge from the Google Play Store or www.bacticount.com. The main screen offers a choice to "Start Bacterial or Viral Analysis" and the user is prompted to pick the sample type; i.e., blood, urine, feces, or saliva (**eFigure 2**). The user follows a three-step procedure: 1) Record Standard Curve for a pathogen of interest in contrived (spiked) samples (e.g., SARS-CoV-2; influenza); 2) Record Sample; and 3) Select and view results where the app displays the sample results in a binary manner as follows: "Pathogen Detected" - designated as red circle; or, "No Pathogen Found" - designated as green circle on the "Reaction Results" screen. Further, by clicking on the red circle that appears if a pathogen is detected, the app then displays the viral load in copies/mL on the "Detailed Reaction Results" screen.

Establishing standard curve, unknown sample reactions, and data analysis

When running a sample reaction, the app launches a specialized viewfinder, allowing the user to center the reaction vials in the view-frame of the phone's camera, such that their intensity can be analyzed over time as adapted from [4] for up to 96 sample wells (**eFigure 2**). After entering the sample name, the user loads samples and presses "OK", which starts a timer to measure lost reaction time while setting up the box and aiming the camera. When the user selects "Begin Recording Amplification," the application proceeds to capture one photograph of the amplification reaction every 10 s over the entire course of the allotted reaction time. The user has three options:

1. Record Standard Curve option, the software also prompts the user to align each reference sample with a provided sample map so that the input starting concentrations of nucleic acid are known. The standard curve is determined through a linear regression fit of T_t vs. $\log_{10}$[conc], which is stored as a '.pasc file' for determining the results in future tests.

2. Record Sample option, the app will record traces for each sample. The numerical sample traces and collected time-stamped photos are saved as a '.parr file' and as '.jpeg files', respectively, which may be extracted by the user to any computer.

3. Select and view results option, the app will prompt the user to choose a standard curve that has been recorded as outlined in the previous section with known standard concentrations. After data processing and analysis, the T_t of unknown test samples are related to their initial concentrations via the standard curve. On its final screen, the app displays the viral load for each positive reaction well that was initially scored in a binary manner as either positive (red) or negative (green).

Data analysis. The Bacticount app enables the smartphone to serve as a stand-alone diagnostic for sensitivity (binary +/- call result) and quantitative detection of microbial titers as adapted from Barnes et al.[4] Real-time LAMP traces were automatically generated for each sample and used to calculate the threshold time (Tt) and concentration by the app on the smartphone. The Tt is linearly related to the logarithm of the input concentration and is used to determine the concentration of virus in saliva samples using standard curves. Alternatively, trace files from the phone were transferred to a personal computer (PC), where a custom MATLAB script was used to determine the Tt and calculate the resultant virus concentration using standard curves with Microsoft Excel.

Strains

Coronaviruses: The following reagents were obtained from BEI Resources, NIAID, NIH or ATCC. Inactivated SARS-CoV-2 isolate from USA-WA1/2020 (NR-52286); genomic RNA from SARS-CoV-2, isolate Hong Kong/VM20001061/2020 (NR-52388); genomic RNA from SARS-CoV-1 (NR-52346); and inactivated Middle East Respiratory Syndrome coronavirus MERS-CoV EMC/2012 (NR-50549). Human seasonal coronaviruses HCoV-OC43 (VR-1558), HCoV-229E (VR-740), HCoV-NL63 (NR-470), and genomic RNA from HCoV-HKU1 (VR-3262SD). SARS-CoV-2 variant genomic RNA was obtained from Carolina Arias and Zach Aralis (University of

California, Santa Barbara): Alpha, B.1.1.7 (UK); Gamma, P.1 (Brazil); Delta, B.1.617.2 (India); Epsilon, B.1.429 (CAL20C); and Iota, B.1.526 (NY). Influenza virus strains: influenza A Virus, A/San Diego/1/2009 (H1N1)pdm09 (NR-15241) and influenza B virus, B/Christchurch/33/2004 (Yamagata Lineage) (NR-36526) were obtained from BEI Resources. The viral genomic RNA and virus stocks were aliquoted and stored at -80 °C. Stocks were not thawed and frozen more than three times. Bacterial respiratory pathogens: *S. pneumoniae* D39, *S. aureus* USA 300, *P. aeruginosa* ATCC 10145 and *K. pneumoniae* ATCC 13883 were as previously described.[4,5]

Oligonucleotide Primers

Table S1 lists the oligonucleotide primer sequences used in this study. The smaRT-LAMP primer sets consist of two outer (F3 and B3), two inner (FIP and BIP), and two loop primers (F-Loop and B-Loop). Primers for smaRT-LAMP analysis target the SARS-CoV-2 nucleocapsid (N) and ORF1ab genes; influenza primers target genes encoding matrix protein (M1) and polymerase (PB1) for influenza A; and M1 and nonstructural protein (NS1) for influenza B. SARS-CoV-2 LAMP primer sets targeting ORF1ab and N genes (sets 9 and 16, respectively) were used for all analyses in this study. Alternative SARS-CoV-2 LAMP primers sets targeting ORF1ab and N genes have been validated for smaRT-LAMP (sets 15 and 3, respectively). Primers for CDC 2019-nCoV RT-qPCR analysis targets the SARS-CoV-2 N gene,[6] while the influenza SARS-CoV-2 (Flu SC2) RT-qPCR multiplex assay targets M1 for influenza A; and NS2 for influenza B.[7]

New smaRT-LAMP primer sets (SARS-CoV-2, set 9; influenza A, set A5a; and influenza B, sets B5, B7) were designed as follows. A minimum of 42 genome sequences each for SARS-CoV-2, influenza A, and influenza B were downloaded from the NCBI virus genome database (SARS-CoV-2) or NCBI Influenza Virus Database (influenza A and B), and aligned using CLC sequence viewer (version 6.8.1), to identify conserved regions and consensus target sequences for primer design. Loop primers were designed for SARS-CoV-2 alternative primer set 15 adapted from Ganguli et al.[8] Influenza A primer set A6 was adapted from Poon et al. [9] by designing loop primers and using consensus sequences to redesign primers.

LAMP reagents

Calcein, KCl, $MnCl_2$, $(NH_4)_2SO_4$, and Triton X-100 were purchased from Millipore Sigma (St. Louis, MO). Bst 2.0 WarmStart DNA polymerase, WarmStart RTx reverse transcriptase, isothermal amplification buffer, $MgSO_4$, RNase inhibitor (murine) and deoxynucleotide triphosphates were purchased from New England Biolabs (Beverly, MA); 1M Tris (pH 7.5) from Invitrogen (Carlsbad, CA); and, nuclease-free water, DMSO, NaOH, and polysorbate 20 (Tween-20) from ThermoFisher (Waltham, MA). Eight-tube PCR strips and 96-well PCR plates with optically clear lid strips were purchased from Bio-Rad (Hercules, CA). Oligonucleotide primers were purchased from Integrated DNA Technologies (IDT, Coralville, IA).

RT-qPCR reaction conditions

The CDC 2019-nCoV RT-qPCR [6] and Flu SC2 RT-qPCR [7] tests contains primers and probes for SARS-CoV-2; and SARS-CoV-2, influenza A and B, respectively. SARS-CoV-2 was evaluated using SARS-CoV-2, influenza A, or influenza B primer and probes (IDT) and the LUNA cell-ready probe one step RT-qPCR kit (NEB). RT-qPCR was performed using 10 μL saliva sample as per manufacturer recommendations.

SmaRT-LAMP platform hardware

SmaRT-LAMP hardware was adapted from Barnes et al.[4] Experiments were performed in low-profile 0.2-mL PCR strips (Bio-Rad cat. no. TLS0801) or 96 well PCR plates (Bio-Rad cat. no. MLL9601) covered with optical flat strips (Bio-Rad cat. no. TLS0803). Sample tubes were placed in an aluminum sample block (LightLabs cat. no. A-7079) on a hot plate (HP30A digital aluminum hot plate, Torrey Pines Scientific, Carlsbad, CA). A cardboard box covering the hot plate was painted black and a flexible LED cable of 96 W, 480 nm, 672 lumens, 96-LEDs (DealeXtreme cat. no. 180563) was affixed to the inside top cover of the box to excite the calcein dye at 480 nm. LEDs were powered using a single output DC power supply (UA8001A, Agilent Technologies, Santa Clara, CA). A Samsung Galaxy S7 or S9 smartphone (Samsung Electronics Co., Ltd.) was outfitted with a 520 ± 10 nm bandpass filter (Edmund Optics cat. no. 65-699) for visual detection of emitted green light. All RT-qPCR reactions were performed on a Bio-Rad CFX96 qPCR Thermocycler.

SmaRT-LAMP sensitivity and specificity assays

SmaRT-LAMP can simultaneously detect SARS-CoV-2, influenza A and/or B viruses via addition of cognate primers to individual wells, which is clinically important when both SARS-CoV-2 and influenza viruses are co-circulating as the two disease syndromes are similar[10]. Sensitivity and specificity tests were performed using contrived saliva specimens as recommended under FDA Emergency Use Authorization (EUA) guidelines.[11]

Sensitivity: Limit of detection (LOD) was evaluated following serial dilution of viral and genomic RNA (genome copies/mL) or viral stocks quantified by the 50% tissue culture infective dose assay [$TCID_{50}$, the highest dilution causing a cytopathic effect in one-half of tissue culture samples].[12] Viral pathogens quantified by $TCID_{50}$/mL include: influenza A, 1.4 x 10^5 $TCID_{50}$/mL; influenza B, 8.0 x 10^4 $TCID_{50}$/mL; HCoV-NL63, 8.0 x 10^3 $TCID_{50}$/mL; HCoV-229E, 8.0 x 10^5 $TCID_{50}$/mL; HCoV-OC43: 4.5 x 10^4 $TCID_{50}$/mL. LOD was determined by the largest serial dilution giving a signal in $\geq$ 19/20 biological replicates.

Specificity: Cross-reactivity tests were performed with contrived saliva specimens using EUA recommended amounts of 10^5 and 10^6 copies/mL specimen for viral and bacterial pathogens, respectively; or via undiluted viral stocks quantified by $TCID_{50}$.[11] Specificity was determined by the presence or absence of signal (binary +/- call). n= 10 biological replicates for SARS-CoV-2 variants; n = 20 biological replicates for all other pathogens.

Analysis of virus present in saliva

Spiked saliva. Known amounts of inactivated SARS-CoV-2 as well as other viral and bacterial respiratory pathogens were added to virus-negative human saliva; serial dilutions were made using saliva diluent. Samples were analyzed using both smaRT-LAMP and RT-qPCR to assess specificity and sensitivity of the assay.

Patient saliva. Saliva specimens were collected at Santa Barbara Cottage Hospital of the Cottage Health system, Santa Barbara, CA. Two sub-groups of participants (symptomatic and asymptomatic) were enrolled. The symptomatic group consisted of recruited patients who tested positive for SARS-CoV-2 with symptoms; the asymptomatic patients were recruited from the same community, through negative admission screening tests for SARS-CoV-2 infection. Patient saliva specimens were collected in sterile plastic tubes and stored frozen at - 20 ^oC on site. Upon transport to UC Santa Barbara, frozen specimens were thawed, heat-inactivated at 95 ^oC for 30 min, aliquoted and stored frozen at - 80 ^oC. For processing, saliva samples were thawed on ice and the reaction mixture was assembled at room temperature. Human subjects approval was obtained from the Institutional Human Subjects Use Committee of the University of California, Santa Barbara and the Institutional Review Board of Santa Barbara Cottage Hospital. To assess potential for real-world application to detect virus in patient saliva samples, we carried out a temporal analysis of patient saliva samples stored up to a week at either 4 ^oC or 25 ^oC using smaRT-LAMP.

© 2022 Heithoff DM et al. *JAMA Network Open.*

eTable 1. Oligonucleotide Primer Sequences.

Gene	Pathogen	Primer Set	Primer Sequences	Ref.
N	SARS-CoV-2	3	F3: 5′-AACACAAGCTTTCGGCAG-3′<br>B3: 5′-GAAATTTGGATCTTTGTCATCC-3′<br>FIP: 5′-TGCGGCCAATGTTTGTAATCAG-CCAAGGAAATTTTGGGGAC-3′<br>BIP: 5′-CGCATTGGCATGGAAGTCAC-TTTGATGGCACCTGTGTAG-3′<br>FL: 5′-TTCCTTGTCTGATTAGTTC-3′<br>BL: 5′-ACCTTCGGGAACGTGGTT-3′	[1]
N	SARS-CoV-2	16	F3: 5′-TGGCTACTACCGAAGAGCT-3′<br>B3: 5′-TGCAGCATTGTTAGCAGGAT-3′<br>FIP: 5′-TCTGGCCCAGTTCCTAGGTAGT-CCAGACGAATTCGTGGTGG-3′<br>BIP: 5′-AGACGGCATCATATGGGTTGCA-CGGGTGCCAATGTGATCT-3′<br>FL: 5′-GGACTGAGATCTTTCATTTTACCGT-3′<br>BL: 5′-ACTGAGGGAGCCTTGAATACA-3′	[2]
ORF1ab	SARS-CoV-2	9	F3: 5′-TCGGTGGACAAATTGTCAC-3′<br>B3: 5′-GTAGGCCAGTTTCTTCTCTG-3′<br>FIP: 5′-GAGTCAGCACACAAAGCCAAAAAT-CTGTGCAAAGGAAATTAAGGAG-3′<br>BIP: 5′-TGGTGGAGCTAAACTTAAAGCCT-CACACTTTCTGTACAATCCCTT-3′<br>FL: 5′-ACAAGCTTAAAGAATGTCTGAACAC-3′<br>BL: 5′-TTAGGTGAAACATTTGTCACGC-3′	This study
ORF1ab	SARS-CoV-2	15	F3: 5′-CGGTGGACAAATTGTCAC-3′<br>B3: 5′-CTTCTCTGGATTTAACACACTT-3′<br>FIP: 5′-TCAGCACACAAAGCCAAAAATTTAT-CTGTGCAAAGGAAATTAAGGAG-3′<br>BIP: 5′-TATTGGTGGAGCTAAACTTAAAGCC-CTGTACAATCCCTTTGAGTG-3′<br>FL: 5′-ACAAGCTTAAAGAATGTCTGAACAC-3′<br>BL: 5′-GAATTTAGGTGAAACATTTGTCACG-3′	*Adapted from ref.[8]
M1	Influenza A H1N1, H3N2	A6	F3: 5′-TGGTGCACTTGCCAGTTG-3′<br>B3: 5′-CCAGCCATCTGTTCCATAGC-3′<br>FIP: 5′-TGCTGTGAATCAGCAATCTGTT-ACAGGATGGGAACAGTGACC-3′<br>BIP: 5′-AGACAGATGGCTACTACCACC-CGTAGTGCTAGCCAGCACC-3′<br>FL: 5′-GCACACACTAGACCAAAAGCAGCTT-3′<br>BL: 5′-TCCACTAATCAGGCATGAAAACAG-3′	*Adapted from ref.[9]
PB1	Influenza A H1N1, H3N2	A5a	F3: 5′-ACCAAGACAACATACTGGTG-3′<br>B3: 5′-GCCACAAATCCATAGCGATA-3′<br>FIP: 5′-TCCACTCCTGCTTGTATTCCCT-TCCAATCATCCGACGATT-3′<br>BIP: 5′-AGGACCTGCAAGTTAGTGGGA-GTGAATTCAAATGTCCCTGT-3′<br>FL: 5′-GGTTTGGTGCATTCACTATGAGAGC-3′<br>BL: 5′-ATCAACATGAGCAAAAAGAAGTCCT-3′	This study
M1	Influenza B Yamagata	B5	F3: 5′-TGAAAGCTCAGCGCTACT-3′<br>B3: 5′-TGTTCATAGCTGAGACCATC-3′<br>FIP: 5′-TCGCACAAAGCACAGAGCGTT-CATGTACCTGAATCCTGGAA-3′<br>BIP: 5′-AGCATCACATTCACACAGGGCT-GCATTTCTCGTCTCACTCC-3′<br>FL: 5′-CCTAGTTTTACTTGCATTGA-3′<br>BL: 5′-AGCAGAGCAGCGAGATCTTC-3′	This study
NS1	Influenza B Yamagata	B7	F3: 5′-AAGTCCTTATCAACTCTGCA-3′<br>B3: 5′-GTGCTCTTGACCAAATTGG-3′<br>FIP: 5′-CGATGGCCATCTTCTTCATCCT-ACCAGAGTGGAAGGCTTGT-3′<br>BIP: 5′-CTCAATTCACTCTTCGAGCGTC-ATAAGACTCCCACCGCAG-3′<br>FL: 5′-CACTGTAAGATCATCAGTAGCAACA-3′<br>BL: 5′-TTAATGAAGGACATTCAAAGCC-3′	This study

*Loop primers were designed for SARS-CoV-2 primer set 15.[8] Influenza A primer set A6 was modified from ref.[9] by designing loop primers, and by using consensus sequences to redesign primers.

© 2022 Heithoff DM et al. *JAMA Network Open.*

eTable 2. Evaluation of LAMP Primer Sequences for Nucleotide Mutations Present in SARS-CoV-2 Variants.

Variant*	Alpha B.1.1.7 (UK)		Beta B.1.351 (S. Africa)		Gamma P.1 (Brazil)		Delta B.1.617.2 (India)		Epsilon B.1.429 (CAL.20C)		Iota B.1.526 (NY)		**LAMP Primers
Gene	Nucleotide[13]	Amino acid[14]	Nucleotide[15]	Amino acid[14]	Nucleotide[16]	Amino acid[14]	Nucleotide[17]	Amino acid[14]	Nucleotide[18]	Amino acid[18]	Nucleotide[19]	Amino acid[19]	
5' UTR											C241T		Not present
ORF1ab	C3267T<br>C5388A<br>T6954C<br>11288-11296 (deletion)	T1001I<br>A1708D<br>I2230T<br>SGF 3675-3677 (deletion)	G5230T	K1655N	C3828T<br>A5648C	S1188L<br>K1795Q<br>11288-11296 (deletion)			C241T<br>C1059T<br>T2597C<br>G3037T<br>A12878G<br>C14408T<br>G17014T	T265I<br>I4205V<br>P314L<br>D1183Y	C1059T<br>C3037T<br>T9867C<br>11288-11297 (deletion)<br>C14408T<br>A16500C<br>A20262G	T851I<br>L438P<br>S106del<br>G107del<br>F108del<br>P323L<br>Q88H	Not present
Spike	21765-21770 (deletion)<br>21991-21993 (deletion)<br>A23063T<br>C23271A<br>C23604A<br>C23709T<br>T24506G<br>G24914C	HV 69-70 (deletion)<br>Y144 (deletion)<br>N501Y<br>A570D<br>P681H<br>T716I<br>S982A<br>D1118H	A21801C<br>A22206G<br>G22813T<br>C23664T<br>A23063T<br>G23012A	D80A<br>D215G<br>K417N<br>A701V<br>N501Y<br>E484K	C21614T<br>C21621A<br>C21638T<br>G21974T<br>G22132T<br>A22812C<br>G23012A<br>A23063T<br>C23525T<br>C24642T	L18F<br>T20N<br>P26S<br>D138Y<br>R190S<br>K417T<br>E484K<br>N501Y<br>H655Y<br>T1027I	C21618G<br>T22917G<br>C22995A<br>C23604G<br>G24410A	T19R<br>L452R<br>T478K<br>P681R<br>D950N	G21600T<br>G22018T<br>T22917G<br>A23403G<br>T24349C	S13I<br>W152C<br>L452R<br>D614G	C21575T<br>C21846T<br>A22320G<br>G23012A<br>A23403G<br>C23664T	L5F<br>T95I<br>D253G<br>E484K<br>D614G<br>A701V	Not present
ORF3a						G174C	C25469T	S26L	G25563T	O57H	C25517T<br>G25563T	P42L<br>Q57H	Not present
E			C26456T	P71L									Not present
M							T26767C	I82T	C26681T				Not present
ORF7a							T27638C<br>C27752T	V82A<br>T120I					Not present
ORF8	C27972T<br>G28048T<br>A28111G	Q27stop<br>R52I<br>Y73C			G28167A	E92K			G27890T (intergenic)		C27925T	T11I	Not present
N	28280 GAT -> CTA<br>C28977T	D3L<br>S235F	C28887T	T205I	C28512G	P80R	A28461G<br>G28881T<br>G29402T	D63G<br>R203M<br>D377Y	A28272T (intergenic)<br>C28887T	T205I	28274del<br>C28869T<br>G28975A	P199L<br>M234I	Not present
Stem Loop											G29764T		Not present

*Variant WHO designation: Alpha, B.1.1.7 (UK); Beta, B.1.351 (S. Africa); Gamma, P.1 (Brazil); Delta, B.1.617.2 (India); Epsilon, B.1.429 (CAL20C); and Iota, B.1.526 (NY).[20]

**Primer set 3 (N; nt 29083 to 29311), set 9 (ORF1ab; nt 2244 to 2453), set 15 (ORF1ab; nt 2245-2441), and set 16 (N; nt 28525 to 28741).

**eTable 3.** SmaRT-LAMP Test expenditures, Equipment and Scale-up Protocol for Reaction Mix.							
**(a) Chemical Reagents**	Volume/ reaction (μL)	Vendor	Part no.	Item Price (USD)	Price/μL (USD)	Price/ Reaction (USD)	Website link
NEB 10X isothermal amplification buffer	5	NEB	M0538M	Comes with Bst			
NEB $MgSO_4$ (100 mM)	1.85	NEB	M0538M	Comes with Bst			
NEB dNTP mix (10 mM)	7	NEB	N0447L	5 vials of 800 μL, 10 mM each nt, $204.00	0.051	0.357	https://www.neb.com/products/n0447-deoxynucleotide-dntp-solution-mix#Product%20Information
FIP/BIP primers (300 μM) for 2 targets	0.261 x 4	IDT	Custom	$0.149314657/μL	0.149314657	0.155884502	
F3/B3 primers (30 μM) for 2 targets	0.34 x 4	IDT	Custom	$0.007475021/μL	0.007475021	0.010166028	
FL/BL primers (30 μM) for 2 targets	1.32 x 4	IDT	Custom	$0.0100047/μL	0.0100047	0.052824818	
NEB Bst 2.0 WarmStart DNA polymerase (120 U/μL)	0.266	NEB	M0538M	8,000 U at 120,000 U/mL, $255.20	3.828	1.018248	https://www.neb.com/products/m0538-bst-20-warmstart-dna-polymerase
NEB RTx WarmStart reverse transcriptase (15 U/μL)	2	NEB	M0380L	15,000 U/mL, 250 rxns (0.5 μL/rxn), $221.60	1.7728	3.5456	https://www.neb.com/products/m0380-warmstart-rtx-reverse-transcriptase#Product%20Information
Fluorescence Detection Reagent (FDR): 0.5 mM calcein, 10 mM $MnCl_2$ in H_2O	2	In house			5.53237E-05	0.000110647	
RNase Inhibitor, Murine	1.25	NEB	M0314L	40,000 U/mL, 15,000 Units, $233.60	0.622933333	0.778666667	https://www.neb.com/products/m0314-rnase-inhibitor-murine#Product%20Information
40% Tween 20	0.5	In house			0.0000446	0.0000223	
40 mM Tris	25	In house			2.3024E-06	0.00005756	

Nuclease-free H_2O	5	Ambion (Thermo)	AM9938	100 mL, $36.03	0.0003603	0.00 180 15	https://www.thermofisher.com/order/catalog/product/AM9938#/AM9938
**Total Price of Chemical Reagents:**						**5.92**	
**(b) Consumables**							
96-well PCR plate	1 of 96 tubes	Thermo (Fisher)	AB0700	25 plates, $27.70	NA	0.01 154 166 7	https://www.thermofisher.com/order/catalog/product/AB0700#/AB0700
Optical flat 8-cap strips	1 of 96 lids	Bio-Rad	TCS0803	128, 8-cap strips, $27.52	NA	0.02 687 5	https://www.bio-rad.com/en-us/sku/tcs0803-0-2-ml-flat-pcr-tube-8-cap-strips-optical-ultraclear?ID=tcs0803
10 µL pipette tips	1 tip	Eppendorf	30078519	960 tips, $131.17	NA	0.13 663 541 7	
100 µL pipette tips	1 tip	Eppendorf	30078551	960 tips, $123.88	NA	0.12 904 166 7	
50 mL Falcon Tubes	1 tube	Corning	1495949 A	500 tubes, $87.98	NA	0.17 596	https://www.fishersci.com/shop/products/falcon-50ml-conical-centrifuge-tubes-25-rack/1495949a
RNase Away	Variable	Thermo	2123621	$21.21 each bottle	NA		https://www.fishersci.com/shop/products/molecular-bioproducts-rnase-away-surface-decontaminant-surface-decontaminant-8-5-oz-bottle-0-25l/2123621
**Total Price of Consumables:**						**0.48**	
**(c) Other calculations**							
Calcein powder (for 50 mM Calcein stock)		Sigma	C0875	5 g, $107.64	0.0006701 67		https://www.sigmaaldrich.com/catalog/product/sigma/c0875?lang=en&region=US
DMSO (for 100 µL working FDR stock)		Thermo Fisher	TS-20684	50 mL, $72.31	0.0014462		https://www.fishersci.com/shop/products/thermo-scientific-silylation-grade-solvents-dimethylsulfoxide-dmso-50ml/pi20684
$MnCl_2$ (for 100 µL working FDR stock)		Sigma	M1787	10 mL, 1M, $34.16	0.003416		https://www.sigmaaldrich.com/catalog/product/sigma/m1787?lang=en&region=US
Calcein stock (50 mM = 31.13 mg/mL DMSO)		In house			0.0055323 67		
Fluorescence Detection Reagent cost		In house		$0.01 for 100 µL FDR	5.53237E-05		
Tween 20 (40% stock)		Fisher	BP337	$11.15/100 mL at 100% stock	0.0000446		https://www.fishersci.com/shop/products/tween-20-fisher-bioreagents-2/BP337100

Tris-HCl 1M, pH 7.5		Invitrogen (Thermo)	15567027	$57.56/L at 1M	0.00005756		https://www.thermofisher.com/order/catalog/product/15567027?us&en#/15567027?us&en
**Price per reaction: 20 µL saliva into 50 µL rxn, 2x Bst, 2x RTx, 1x RI**						**6.40**	
**(d) Equipment: Validated Platform**							
Digital Hot Plate		Torrey Pines Scientific	HP30A	1,260.00			https://www.torreypinesscientific.com/product/digital-standard-hot-plate/
Cardboard Display Board (For Box Construction)		Walmart	730318	3.18			https://www.walmart.com/ip/Elmer-s-Tri-Fold-Self-Standing-Project-Display-Board-36-X-48-Black-1-count/16817689
DC Power Supply		Keysight (previously Agilent)	U8001A	466.00			https://www.keysight.com/us/en/products/dc-power-supplies/bench-power-supplies/u8000-series-bench-power-supply-90-150w.html
480 nm LED lights		Shopmadeinchina (previously DealExtreme)	180563	10.53			http://www.shopmadeinchina.com/product/96W-480nm-672lm-96-LED-Blue-Light-Car-Chassis_14238925.shtml
520 nm green light filter		Edmund Optics	65-699	135.00			https://www.edmundoptics.com/p/520nm-cwl-10nm-fwhm-25mm-mounted-diameter/20217/
96-Well Aluminum Block Sample Holder		LightLabs	A-7079	69.90			https://www.lightlabsusa.com/96-Well-Aluminum-Block.html
**Total Price of Validated Platform:**				**1944.61**			
**(e) Equipment: Low-Cost Alternative Platform**							
Hot Plate (electric single burner with temperature knob)		Amazon	GAU-80305	11.99			https://www.amazon.com/GAU-80305-Electric-Single-Burner-1100-Watts/dp/B005T0SN0K
Cardboard Display Board (for box construction)		Walmart	730318	3.18			https://www.walmart.com/ip/Elmer-s-Tri-Fold-Self-Standing-Project-Display-Board-36-X-48-Black-1-count/16817689
DC Power Supply (9V battery)		BatteryJunction	1222	0.72			https://www.batteryjunction.com/energizer-1222.html
480 nm LED lights		Shopmadeinchina (previously DealExtreme)	180563	10.53			http://www.shopmadeinchina.com/product/96W-480nm-672lm-96-LED-Blue-Light-Car-Chassis_14238925.shtml

520 nm green light filter		AliExpress	SLB520	3.51			https://www.aliexpress.com/item/32900291133.html?aff_platform=portals-tool&sk=_dZqrWKC&aff_trace_key=3b742b01a6d64307a781b867799dbabb-1607110278738-03064-_dZqrWKC&dp=_dZqrWKC-32900291133&terminal_id=d0068eb438984ed581059c768e27a5da&tmLog=new_Detail
96-Well Aluminum Block Sample Holder		LightLabs	A-7079	69.90			https://www.lightlabsusa.com/96-Well-Aluminum-Block.html
**Total Price of Low-Cost Alternative Platform**				99.83			

**(f) Scale-up Protocol for Reaction Mix (96 reactions)***			
	Volume/1 reaction (µL)		Volume/96 reactions (µL)
<u>Master mix components</u>			
NEB 10X isothermal amplification buffer	5		530
NEB $MgSO_4$ (100 mM)	1.85		196.1
NEB dNTP mix (10 mM)	7		742
FIP/BIP primers (300 µM) for 2 targets	0.26 x 4		27.6 x 4
F3/B3 primers (30 µM) for 2 targets	0.34 x 4		36.0 x 4
FL/BL primers (30 µM) for 2 targets	1.32 x 4		139.9 x 4
NEB Bst 2.0 WarmStart DNA polymerase (120 U/µL)	0.27		28.6
NEB RTx WarmStart reverse transcriptase (15 U/µL)	2		212

Fluorescence Detection Reagent (0.5 mM calcein, 10 mM $MnCl_2$ in H_2O)	2		212
40% Tween 20	0.5		53.0
<u>Sample mix components</u>			
Saliva specimen	20		20 x 96
Tris-HCl pH 7.5 (400 mM)	2.5		265
NEB RNase Inhibitor, Murine (40 U/μL)	1.25		132.5
Nuclease-free water	1.25		132.5

*10% additional reagents

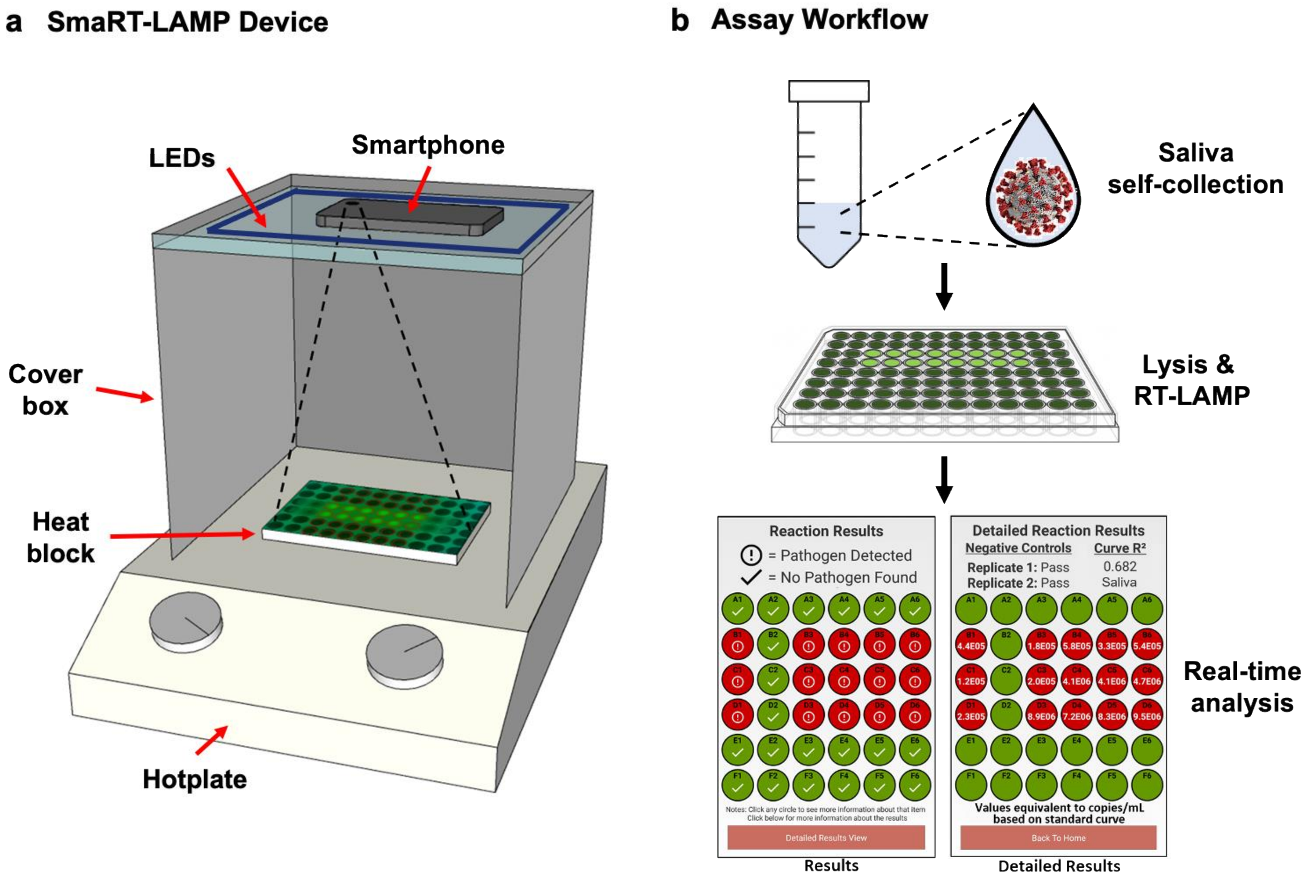

eFigure 1. Overview of SmaRT-LAMP Instrumentation and Workflow. (**a**) Device: LEDs are affixed to the inside top of a cardboard box that covers a heat block resting on a hotplate. A smartphone camera is directed towards the samples through a box aperture. (**b**) Workflow: The smaRT-LAMP reaction mix, containing “sample mix” (saliva specimen with RNA stabilizers) and “master mix” (lysis reagents, primers and polymerase enzymes), is assembled at room temperature and loaded onto a 70 ^{o}C heat block, which initiates both the reverse transcription and LAMP reactions. The mobile phone app displays the sample results in a binary manner as follows: “Pathogen Detected” - designated as red circle; or, “No Pathogen Found” - designated as green circle on the “Reaction Results” screen. Clicking on the red circle results in the app displaying the viral load in copies/mL on the “Detailed Reaction Results” screen.

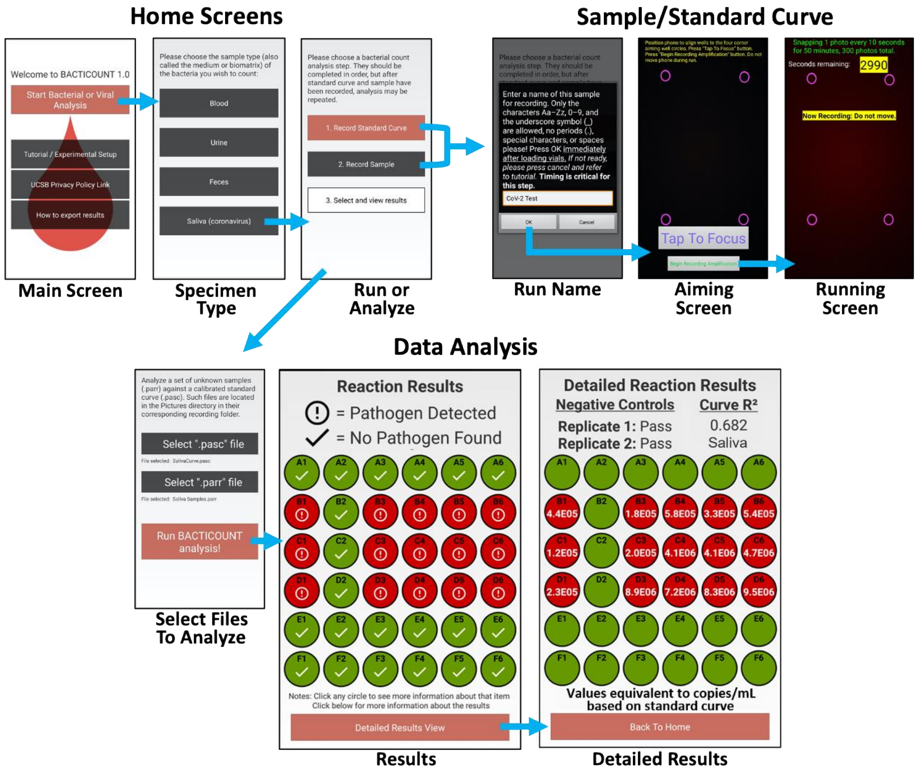

eFigure 2. **Workflow of the Bacticount SmaRT-LAMP Mobile Phone App**. The user is prompted to pick the sample type (saliva) followed by a three-step procedure: 1) Record Standard Curve for a pathogen of interest in contrived (spiked) samples (e.g., SARS-CoV-2; influenza); 2) Record Sample; and 3) Select and view results where the app displays the sample results in a binary manner as follows: "Pathogen Detected" - designated as red circle; or, "No Pathogen Found" - designated as green circle on the "Reaction Results" screen. By clicking on the red circle that appears if a pathogen is detected, the app displays the viral load in copies/mL on the "Detailed Reaction Results" screen. Blue arrows represent connecting app screenshots.

eReferences

1. Broughton J, Deng X, Yu G, et al. CRISPR–Cas12-based detection of SARS-CoV-2. *Nat Biotech.* 2020;38(7):870-874. doi:10.1038/s41587-020-0513-4.
2. Thi V, Herbst K, Boerner K, et al. A colorimetric RT-LAMP assay and LAMP-sequencing for detecting SARS-CoV-2 RNA in clinical samples. *Sci Transl Med.* 2020;12(556):eabc7075. doi:10.1126/scitranslmed.abc7075.
3. Meagher R, Priye A, Light Y, Huang C, Wang E. Impact of primer dimers and self-amplifying hairpins on reverse transcription loop-mediated isothermal amplification detection of viral RNA. *Analyst.* 2018;143(8):1924-1933. doi:10.1039/c7an01897e.
4. Barnes L, Heithoff D, Mahan S, et al. Smartphone-based pathogen diagnosis in urinary sepsis patients. *EBioMedicine.* 2018;36:73-82. doi:10.1016/j.ebiom.2018.09.001.
5. Ersoy S, Heithoff D, Barnes L, et al. Correcting a fundamental flaw in the paradigm for antimicrobial susceptibility testing. *EBioMedicine.* 2017;20:173-181. doi:10.1016/j.ebiom.2017.05.026.
6. Centers for Disease Control and Prevention. CDC's diagnostic test for COVID-19 only and supplies. 2020. https://www.cdc.gov/coronavirus/2019-ncov/lab/virus-requests.html. Accessed June 19, 2021.
7. Centers for Disease Control and Prevention. CDC's influenza SARS-CoV-2 multiplex assay and required supplies. 2021. https://www.cdc.gov/coronavirus/2019-ncov/lab/multiplex.html. Accessed May 6, 2021.
8. Ganguli A, Mostafa A, Berger J, et al. Rapid isothermal amplification and portable detection system for SARS-CoV-2. *Proc Natl Acad Sci USA.* 2020;117(37):22727-22735. doi:10.1073/pnas.2014739117.
9. Poon L, Leung C, Chan K, et al. Detection of human influenza A viruses by loop-mediated isothermal amplification. *J Clin Microbiol.* 2005;43(1):427-430. doi:10.1128/JCM.43.1.427-430.2005.
10. Centers for Disease Control and Prevention. Testing guidance for clinicians when SARS-CoV-2 and influenza viruses are co-circulating. 2021. https://www.cdc.gov/flu/professionals/diagnosis/testing-guidance-for-clinicians.htm. Accessed May 4, 2021.
11. U.S. Food and Drug Administration. Molecular diagnostic template for laboratories. 2021. https://www.fda.gov/medical-devices/coronavirus-disease-2019-covid-19-emergency-use-authorizations-medical-devices/in-vitro-diagnostics-euas. Accessed June 19, 2021.
12. Klimov A, Balish A, Veguilla V, et al. Influenza virus titration, antigenic characterization, and serological methods for antibody detection. *Influenza Virus.* 2012:25-51. doi:10.1007/978-1-61779-621-0_3.
13. Rambaut A, Loman N, Pybus O, et al. Preliminary genomic characterisation of an emergent SARS-CoV-2 lineage in the UK defined by a novel set of spike mutations. 2020. https://virological.org/t/preliminary-genomic-characterisation-of-an-emergent-sars-cov-2-lineage-in-the-uk-defined-by-a-novel-set-of-spike-mutations/563. Accessed February 15, 2021.
14. O'Toole A, Scher E, Underwood A, et al. Pangolin: lineage assignment in an emerging pandemic as an epidemiological tool. 2021. https://cov-lineages.org/global_report.html Accessed Febrary 15, 2021.
15. Tegally H, Wilkinson E, Giovanetti M, et al. Detection of a SARS-CoV-2 variant of concern in South Africa. *Nature.* 2021;592(7854):438-443. doi:10.1038/s41586-021-03402-9.
16. Naveca F, Nascimento V, Souza V, et al. Phylogenetic relationship of SARS-CoV-2 sequences from Amazonas with emerging Brazilian variants harboring mutations E484K and N501Y in the Spike protein. 2021. https://virological.org/t/phylogenetic-relationship-of-sars-cov-2-sequences-from-amazonas-with-emerging-brazilian-variants-harboring-mutations-e484k-and-n501y-in-the-spike-protein/585. Accessed February 15, 2021.
17. Arias C. Santa Barbara County Community Data Dashboard. 2021. https://experience.arcgis.com/experience/030e625c69a04378b2756de161f82ef6. Accessed June 11, 2021.

18. Deng X, Garcia-Knight M, Khalid M, et al. Transmission, infectivity, and antibody neutralization of an emerging SARS-CoV-2 variant in California carrying a L452R spike protein mutation. *medRxiv.* 2021;https://www.medrxiv.org/content/10.1101/2021.03.07.21252647v1
19. Cadena J, Muñoz M, León G, et al. Detection of the new SARS-CoV-2 variant B. 1.526 with the Spike E484K mutation in South America. 2021. https://www.researchsquare.com/article/rs-248965/v1. Accessed May 7, 2021.
20. World Health Organization. Tracking SARS-CoV-2 variants. 2021. https://www.who.int/en/activities/tracking-SARS-CoV-2-variants. Accessed June 10, 2021.
